# Sex education and self-poisoning in Sri Lanka: an explorative analysis

**DOI:** 10.1186/s12889-021-12374-4

**Published:** 2022-01-06

**Authors:** Grace Crowley, Piumee Bandara, Lalith Senarathna, Ayodhya Malalagama, Sonali Gunasekera, Thilini Rajapakse, Duleeka Knipe

**Affiliations:** 1 Population Health Sciences, Bristol Medical School, University of Bristol, Bristol, UK; 2 South London and Maudsley NHS Foundation Trust, London, UK; 3 South Asian Clinical Toxicology Research Collaboration, Faculty of Medicine, University of Peradeniya, Peradeniya, Sri Lanka; 4 Translational Health Research Institute, Western Sydney University, New South Wales, Australia; 5 Department of Health Promotion, Faculty of Applied Sciences, Rajarata University of Sri Lanka, Mihintale, Sri Lanka; 6 Mental Health Unit, Bundaberg Hospital, Bundaberg, Queensland Australia; 7 Family Planning Association of Sri Lanka, Colombo, Sri Lanka; 8 Department of Psychiatry, Faculty of Medicine, University of Peradeniya, Peradeniya, Sri Lanka

**Keywords:** Self-poisoning, Self-harm, Suicidal behaviour, Sex education, Sri-Lanka

## Abstract

**Background:**

Self-harm and suicide are important causes of morbidity and mortality in Sri Lanka, but our understanding of these behaviours is limited. Qualitative studies have implicated familial and societal expectations around sex and relationships. We conducted an explorative analysis using case-control data to investigate the association between sex education and self-poisoning in Sri Lanka.

**Methods:**

Cases (*N*=298) were self-poisoning inpatients on a toxicology ward, Teaching Hospital Peradeniya. Controls (*N*=500) were sex and age frequency matched to cases and were outpatients/visitors to the same hospital. Participants were asked whether they had received sex education, and to rate the quality and usefulness of any sex education received. Logistic regression models adjusted for age, sex, and religion quantified the association between receipt, quality and usefulness of sex education and self-poisoning. We tested whether the associations differed by sex.

**Results:**

Roughly 1-in-3 cases and 1-in-5 controls reported having not received sex education. Individuals who did not receive sex education were nearly twice as likely to have self-poisoned than those who did (OR 1.68 (95% CI 1.11-2.55)). Those who reported the sex education they received as not useful were more likely to have self-poisoned compared to those who reported it useful (OR 1.95 (95% CI 1.04-3.65)). We found no evidence of an association between self-poisoning and the self-rated quality of sex education, or that associations differed by participant sex.

**Conclusion:**

As sex education is potentially modifiable at the population-level, further research should aim to explore this association in more depth, using qualitative methods and validated measurement tools.

**Supplementary Information:**

The online version contains supplementary material available at 10.1186/s12889-021-12374-4.

## Background

It is estimated that 77% of all suicides occur in low- and middle-income countries (LMICs) [1]. Sri Lanka is a middle-income country that has seen a dramatic decrease in suicide rates since the 1990s, coinciding with the banning of the most toxic pesticides in the country [2]. However, the rates of non-fatal self-poisoning over this time period have increased [3] and self-poisoning remains an important cause of morbidity and mortality in the country [4]. Self-poisoning is the most frequent method of hospital-presenting self-harm in Sri Lanka and other methods such as cutting are comparatively uncommon [5]. Mental illness and history of self-harm are considered important risk factors for suicidal behaviour in high-income countries (HICs) [6, 7] but evidence suggests these are likely to play a less significant role in suicidal behaviour in Sri Lanka and other LMICs [8–10]. In Sri Lanka, financial, family and relationship factors are likely to be stronger determinants [11, 12]. Evidence from qualitative studies suggests that relationship factors such as unwanted pregnancies, love affairs, unhappy sexual relationships and sexual assault are frequently implicated in suicide attempts [12]. Self-harm among young women has been described as a form of “dialogue”, sometimes as a way to register moral claims about themselves or others when judged to have engaged in socially unacceptable behaviour [13]. Conflicts between parents and their children regarding sexual relationships have been identified as potential triggers for suicidal behaviour, especially among young people who have engaged in behaviour perceived to bring shame upon themselves and their family [12–15]. Furthermore, it has been suggested that lesbian, gay, bisexual, transgender, queer + (LGBTQ+) individuals may be at increased risk of suicidal ideation [16], as they remain highly stigmatised and criminalized in Sri Lanka, and are also likely to be seen as non-conforming of strict gender and heterosexual norms.

Strict gender norms around sexual behaviour exist for both men and women in Sri Lanka, however girls in particular are expected to conform to sexual modesty [17]. Premarital sex is widely condemned, and strong emphasis is placed on preserving the virginity of unmarried women and girls, meaning the movements of females are monitored to a great degree by their families [14, 18, 19]. Pregnancy out of wedlock, or even expressing knowledge of sex before marriage, may be sufficient to threaten the reputation of a woman and her family, and lead to consequences such as being ostracised from society, removal from the family home and consideration or attempt of suicide [17, 20–22]. These strong ideas about respectability and virtue have been linked to ‘suicide-like acts’, especially among girls and young women [12–14].

Anthropological work has provided insights into the cultural taboos surrounding relationships and sex in Sri Lanka [17, 19]. Sex is a topic that is not openly discussed. Parents may feel embarrassed at the thought of teaching their children about sex, and think that teachers, doctors, peers or magazines are more appropriate sources of such information [19, 23]. The internet is an increasing source of sex education for children and young people across the world [24], and may be an invaluable source of information for those who are not able to talk openly about sex within their community. However, the availability of the internet remains an issue for many young people in Sri Lanka [25, 26], and it is likely that much of what young people learn about sex online is in the form of pornography as opposed to information on sexual health or healthy relationships. In an environment where sex is not openly discussed at home, and reliable information from the internet may not be available, delivering good quality sex education in schools becomes even more important. As Sri Lanka has one of the highest school enrolment rates in South Asia, there is an opportunity for widespread coverage [27, 28].

Traditionally, the main aims of sex education have been to prevent unwanted pregnancies and reduce sexually transmitted infections, but there is increasing evidence that sex education may have broader positive implications. A recent systematic review found that school-based sex education programmes have myriad benefits for the lives of children and young people, such as reduction in domestic violence, prevention of child sexual abuse and development of healthy relationships [29]. However, to date there has been a lack of research examining possible associations between school-based sex education and suicidal ideation or behaviour. One study from the United States (US) found an association between LGBTQ+-inclusive sex education and reduced odds of reporting suicidal thoughts and plans among all youth [30]. The majority of sex education research originates from HICs, predominantly the US, and further work is needed to determine the breadth of benefits of school-based sex education in LMICs.

In keeping with greater understanding of the range of potential benefits of sex education, the World Health Organisation (WHO) promotes the concept of Comprehensive Sexuality Education (CSE) [31]. In Sri Lanka, most of the key components of CSE are included in laws and policies [32] and sex education is part of the national secondary school curriculum [24]. However, this does not necessarily translate into practice, and the sex education currently delivered largely consists of the anatomical and physiological components of reproduction, while neglecting important issues around relationships and gender rights. Sexual and reproductive health knowledge among adolescents has been identified as poor [26, 33, 34]. For example, a survey of 2020 pupils aged 16-19 years found that only 1 in 10 could correctly name a method of contraception [34]. Potential reasons for this are lack of teacher training and a sense of shyness or embarrassment among teachers [35, 36], reflecting attitudes prevalent in society more generally. Furthermore, the belief that sex education will encourage sexual activity among young people is also common among both teachers and parents [35]. This was demonstrated when a recent attempt by the Ministry of Education and the Ministry of Health to introduce a Grade 7 sex education textbook was met with public outrage [37].

Existing knowledge paints a picture of a milieu in which sex is tabooed, sexual behaviours impact profoundly on one’s place in society, and sex and relationship issues have been implicated in acts of self-harm. Sex education is a modifiable intervention that may be used to introduce young people to topics such as gender rights, healthy relationships and emotional wellbeing [31]. To our knowledge, no studies have formally explored the association between self-poisoning and sex education, in any context.

Our aim was to explore whether there is an association between sex education and self-poisoning in Sri Lanka. In this explorative study, we investigated whether individuals who self-poisoned were more likely to report: i) not receiving formal sex education, and that any sex education received was ii) poor quality and iii) not useful, compared to controls. We also tested to see whether any associations were stronger for females than males.

## Methods

### Study design and setting

This is a secondary analysis of data collected for a hospital-based case-control study that primarily sought to examine the association between self-poisoning and adverse childhood experiences in Sri Lanka. The protocol and results of the primary study have been published elsewhere [38, 39].

Sri Lanka is an island in South Asia and has a school enrolment rate of close to 100% [27]. The study took place in the district of Kandy which has a population of approximately 1.4 million [40]. Demographics in the Kandy district closely reflect those of the national population, with Sinhalese being the majority ethnicity (Kandy 74%, national 75%) and Buddhism the main religion (Kandy 73%, national 70%) [40].

### Participant recruitment

Cases (*n*=298) were drawn from patients aged 18 years or over who were admitted to the Toxicology ward of the Teaching Hospital Peradeniya (THP) for the medical management of self-poisoning from 18^th^ July - 31^st^ December 2018. Controls were recruited using age and sex frequency matching. Hospital-based controls (*n*=526) were drawn from patients or visitors to the outpatient department or clinics of THP. Recruited outpatients presented with conditions that were unrelated to the outcome of interest of the study, such as cough, chest infection and hypertension. The study was designed as a hospital-based case-control study, however it was subsequently decided to recruit a community control series to address potential selection bias. Community controls (*n*=480) were recruited from 19^th^ January - 2^nd^ April 2019 by study researchers going door to door in 12 villages, which were randomly selected from a total of 159 within two divisional secretariat areas in Kandy district. Demographics of these villages were compared to 2017 census data to ensure community controls were similar to the source population in terms of sex, age and ethnicity.

Cases and controls were excluded from the study if they were: (i) physically unable or too unwell to participate and/or (ii) had a pre-existing diagnosis of intellectual disability or dementia. Controls were excluded if they reported a history of self-harm, regardless of the method/s used, time since last episode, or intent (hospital controls: *n*=26, 5%).

### Data collection

Data were collected during face-to-face, individual, structured interviews, which were conducted by trained interviewers in the participant’s preferred language (Sinhala, Tamil or English). Interviews were conducted in a private setting to ensure confidentiality and participant safety. Interviewers used a standardised script for cases and controls and were trained to deliver the questions in a standardised manner. A protocol for ensuring participant safety was utilised, which included referral and support for participants who expressed suicidal thoughts and/or domestic violence (further details are provided here [38]).

### Measures

Information collected during the interviews included; demographics, family factors (including number of children), childhood adversity (using the Adverse Childhood Experiences international questionnaire, which included childhood sexual abuse [41]), domestic abuse (using the Humiliation, Afraid, Rape, Kick questionnaire [42]), school connectedness and social capital. The schedule also included questions related to sex education, which were suggested by representatives of a family planning non-governmental organisation (NGO) following community consultation workshops [15]. The questions included *“Did you learn about sexual and reproductive health and rights in school or through another source?”* (yes/no), *“If through school, do you think the information you received was sufficient and was effectively delivered”* (yes/no) and *“Was the information useful to you?”* (yes/no). These were labelled as “receipt”, “quality” and “usefulness” of sex education, respectively. The quality and usefulness questions were re-coded separately into three-point responses to include those who did not receive sex education, as follows; *“did not receive sex education”, “poor quality/not useful sex education”* and *“good quality/useful sex education”.*

### Statistical analysis

A statistical analysis plan was published on the Open Science Framework prior to conducting the main analyses [43]. Stata-15 was used for all statistical analyses [44]. Consistent with previous analyses of this case-control study [39, 45], the primary analyses used the hospital control series. A sensitivity analysis was performed using the community control series to establish whether there was selection bias in the hospital control series and is reported in the Supplementary Tables. The distribution of exposure variables (*receipt, quality and usefulness of sex education*) and confounders (*sex, age, religion, marital status* and *highest educational attainment of either parent)* was reported by case-control status. Religion was included in the models as a dichotomous variable to reflect majority (Buddhist) and minority (non-Buddhist) religions.

Unconditional logistic regression was used to examine the associations between self-poisoning and the three main sex education exposures of interest (*receipt, quality and usefulness of sex education*). This differs from the original protocol where we specified a matched analysis (i.e., conditional logistic regression). We deviated from the protocol to increase the statistical precision of the study without losing validity [46]*.* The hypothesised lowest-risk category in each variable was used as the reference group. Age- and sex-adjusted estimates (the equivalent of ‘crude’ associations for a matched case-control study) were reported in Supplementary Table 1. Model 1 was adjusted for known confounders of the association between sex education and self-poisoning; *sex, age* and *religion.* This was the primary regression model that formed the basis of the study’s conclusions. A second model (Model 2) was run, adjusting for the potential confounder of *highest educational attainment of either parent*, as well as the confounders in Model 1. Both models were then stratified by sex and formally tested for interaction by adding an interaction term. We did not consider participants’ educational attainment as a confounder as it was felt this would sit on the causal pathway, i.e., not receiving schooling would likely lead to lack of sex education in this context.

Two further variables were considered as potentially related to both sex education and self-poisoning (*perception of teachers’ interest in them* and *perception of their parents’ understanding of their concerns*). However, these were considered as potentially sitting on the causal pathway of this association, therefore we report the distribution in cases and controls but do not adjust for them in regression analyses.

Data were available on three further variables that were considered as potentially sitting on the causal pathway between sex education and self-poisoning; *number of children, past-year domestic violence* and *childhood* *sexual abu*se. A formal mediation analysis was not performed given the exploratory nature of this study. However, a table reporting the distribution of these variables stratified by both receipt of sex education and case-control status was included in the Supplementary Tables, for comment in the discussion.

### Missing data

Missing data were reported as numbers and percentages for all variables. Complete cases and controls were used for the logistic regression analyses, i.e., only those participants with no missing data were included. A sensitivity analysis was performed using all available data and is included in the Supplementary Tables.

### Post-hoc analysis

As a post-hoc analysis, we also fitted a third regression model (Model 3) which adjusted for the confounders in Model 1 (age, sex and religion) plus marital status. This was decided following discussions between study authors, during which new background information came to light regarding an educational scheme in Sri Lanka for newly married couples that is coordinated by the divisional Medical Officer of Health departments [47]. As the questions used to ascertain receipt and usefulness of sex education did not discriminate between sex education received at school or through another source, it was thought married participants may have reported on family planning/sex education received through this scheme when answering these questions.

## Results

In total, 298 cases of self-poisoning and 500 hospital controls were recruited. The distribution of demographics and other study variables among cases and controls are reported in Table 1. Among those who had self-poisoned, just over a third had not received any sex education (34.9% (95% CI 29.7-40.5)), compared to 21.6% (95% CI 18.2-25.4) of controls (Table 1). A similar proportion of cases and controls who had received sex education reported it was good quality (cases: 43.4% (95% CI 37.8-49.0), controls: 45.2% (95% CI 40.9-49.6)). However, controls were more likely to rate the sex education they received as useful (72.4% (95% CI 68.3-76.1)) compared to cases (56.4% (95% CI 50.7-61.9)).

**Table 1:** Distribution of study variables among cases and controls (Total *N* = 798)

	Cases n, % (95% CI) *N* = 298	Hospital controls n, % (95% CI) *N* = 500
**Sex**		
Male	142, 47.7 (42.0-53.3)	205, 41.0 (36.8-45.4)
Female	156, 52.3 (46.7-58.0)	295, 59.0 (54.6-63.2)
Missing	0, 0.0	0, 0.0
**Age**		
Median (IQR)	26.0 (21.0-37.0)	26.0 (21.0-36.0)
Missing	0, 0.0	0, 0.0
**Religion**		
Buddhist	225, 75.5 (70.3-80.1)	448, 89.6 (86.6-92.0)
Hindu	35, 11.7 (8.5-15.9)	17, 3.4 (2.1-5.4)
Muslim	21, 7.0 (4.6-10.6)	24, 4.8 (3.2-7.1)
Christian	17, 5.7 (3.6-9.0)	11, 2.2 (1.2-3.9)
Missing	0, 0.0	0, 0.0
**Highest educational attainment from either parent**		
No schooling	17, 5.7 (3.4-9.0)	7, 1.4 (0.7-2.9)
Completed grades 1-10	105, 35.2 (30.0-40.8)	154, 30.8 (26.9-35.0)
Passed O/L	56, 18.8 (14.7-23.6)	140, 28.0 (24.2-32.1)
Passed A/L or completed university/postgraduate qualifications	61, 20.5 (16.3-24.4)	160, 32.0 (28.1-36.2)
Missing	59, 19.8 (15.7-24.7)	39, 7.8 (5.7-10.5)
**Marital status**		
Married/living with partner	157, 52.7 (47.0-58.3)	234, 46.8 (42.5-51.2)
Single	122, 40.9 (35.5-46.6)	257, 51.4 (47.0-55.8)
Divorced/separated/widowed	19, 6.4 (4.1-9.8)	9, 1.8 (0.9-3.4)
Missing	0, 0.0	0, 0.0
**Extent of agreement that teachers at school were interested in them and their life, not just their schoolwork**		
Agree strongly	162, 54.4 (48.7-59.9)	261, 52.2 (47.8-56.6)
Agree somewhat	41, 13.8 (10.3-13.2)	101, 20.2 (16.9-24.0)
Neither agree/disagree	35, 11.7 (8.5-15.9)	44, 8.8 (6.6-11.6)
Disagree somewhat	23, 7.7 (5.2-11.4)	29, 5.8 (4.1-8.2)
Disagree strongly	31, 10.4 (7.4-14.4)	52, 10.4 (8.0-13.4)
Missing	6, 2.0 (0.9-4.4)	13, 2.6 (1.5-4.4)
**Extent to which parents/guardians understood their problems and worries during their first 18 years of life**		
Parents did understand their problems and worries	27, 9.1 (6.3-12.9)	8, 1.6 (0.8-3.2)
Parents did not understand their problems and worries	266, 89.3 (85.2-92.3)	488, 97.6 (95.8-98.6)
Missing	5, 1.7 (0.7-4.0)	4, 0.8 (0.3-2.1)
**Receipt of sex education**		
Yes	191, 64.1 (58.5-69.3)	390, 78.0 (74.1-81.4)
No	104, 34.9 (29.7-40.5)	108, 21.6 (18.2-25.4)
Missing	3, 1.0 (0.3-3.1)	2, 0.4 (0.1-1.6)
**Quality of sex education received through school**		
Good quality	129, 43.3 (37.8-49.0)	226, 45.2 (40.9-49.6)
Poor quality	59, 19.8 (15.7-24.7)	149, 29.8 (25.9-34.0)
No sex education	104, 34.9 (29.7-40.5)	108, 21.6 (18.2-25.4)
Missing	6, 2.0 (0.9-4.4)	17, 3.4 (2.1-5.4)
**Usefulness of sex education**		
Useful	168, 56.4 (50.7-61.9)	362, 72.4 (68.3-76.1)
Not useful	23, 7.7 (5.2-11.4)	26, 5.2 (3.6-7.5)
No sex education	104, 34.9 (29.7-40.5)	108, 21.6 (18.2-25.4)
Missing	3, 1.0 (0.3-3.1)	4, 0.8 (0.3-2.1)

*A/L* Advanced level

*CI* Confidence interval

*IQR* Interquartile range

*O/L* Ordinary level

After removing 117 participants with missing data, 681 (236 cases, 445 controls) were included in the complete case analyses. The results of the primary multivariable logistic regression model (Model 1) are presented in Table 2. Unadjusted associations are presented in Supplementary Table 1, as per best practice guidelines [48]. There was strong evidence to suggest that those who had not received any sex education were 68% more likely to have self-poisoned than those who had received sex education (OR 1.68 (95% CI 1.11-2.55)). There was no statistical evidence that those who had not received sex education or those who received poor quality sex education were more likely to have self-poisoned than those who received good quality sex education (Table 2). There was statistical evidence to suggest that those who had not received sex education (OR 1.79 (95% CI 1.17-2.72)) and those who received sex education that was not useful (OR 1.95 (95% CI 1.04-3.65)) were more likely to have self-poisoned than those who received useful sex education.

**Table 2:** Primary multivariable logistic regression models for associations between sex education and self-poisoning

	Cases n, % (95% CI) *N* = 236	Hospital controls n, % (95% CI) *N* = 445	Model 1 OR (95% CI) *N* = 681
**Receipt of sex education**			
Yes	167, 70.8 (64.6-76.2)	352, 79.1 (25.1-82.6)	1.00
No	69, 29.2 (23.8-35.4)	93, 20.9 (17.4-24.9)	1.68 (1.11-2.55)
**Quality of sex education received through school**			
Good quality	113, 47.9 (41.6-54.3)	211, 47.4 (42.8-52.1)	1.00
Poor quality	54, 22.9 (18.0-28.7)	141, 31.7 (27.5-31.2)	0.78 (0.53-1.16)
No sex education	69, 29.2 (23.8-35.4)	93, 20.9 (17.4-24.9)	1.54 (0.99-2.38)
**Usefulness of sex education**			
Useful	146, 61.9 (55.5-67.9)	328, 73.7 (69.4-77.6)	1.00
Not useful	21, 8.9 (5.9-13.3)	24, 5.4 (3.6-7.9)	1.95 (1.04-3.65)
No sex education	69, 29.2 (23.8-35.4)	93, 20.9 (17.4-24.9)	1.79 (1.17-2.72)

Hospital controls, complete case analysis (Total *N* = 681)

Model 1: Adjusted for sex, age and religion

*OR* Odds Ratio (if the OR>1, this suggests that exposed individuals were more likely to have self-poisoned than non-exposed individuals)

*CI* Confidence Interval (if the CI overlaps 1, this suggests that there is no statistical evidence of a difference in risk between exposed and non-exposed individuals)

The addition of parental education to the model (Model 2, Tables 3 and 4) did not alter the associations, except for the association between receipt of sex education and self-poisoning, which was attenuated (OR 1.45 (95% CI 0.95-2.23)).

**Table 3:** Secondary multivariable logistic regression models for associations between sex education and self-poisoning

	Model 2 OR (95% CI) *N* = 681	Model 3 OR (95% CI) *N* = 681
**Receipt of sex education**		
Yes	1.00	1.00
No	1.45 (0.95-2.23)	1.69 (1.12-2.57)
**Quality of sex education received through school**		
Good quality	1.00	1.00
Poor quality	0.77 (0.52-1.16)	0.77 (0.52-1.15)
No sex education	1.32 (0.84-2.08)	1.54 (0.99-2.40)
**Usefulness of sex education**		
Useful	1.00	1.00
Not useful	2.17 (1.15-4.11)	1.93 (1.03-3.61)
No sex education	1.55 (1.00-2.39)	1.80 (1.18-2.74)

Hospital controls, complete case analysis (Total *N*=681)

Model 2: Adjusted for sex, age, religion, highest educational attainment of either parent

Model 3: Adjusted for sex, age, religion, marital status

*OR* Odds Ratio (if the OR>1, this suggests that exposed individuals were more likely to have self-poisoned than non-exposed individuals)

*CI* Confidence Interval (if the CI overlaps 1, this suggests that there is no statistical evidence of a difference in risk between exposed and non-exposed individuals)

**Table 4:** Multivariable logistic regression models for associations between sex education and self-poisoning, stratified by sex

	Model 1			Model 2			Model 3		
	Female	Male	*p*	Female	Male	*p*	Female	Male	*p*
	OR (95% CI)	OR (95% CI)		OR (95% CI)	OR (95% CI)		OR (95% CI)	OR (95% CI)	
**Receipt of sex education**									
Yes	1.00	1.00	0.06	1.00	1.00	0.05	1.00	1.00	0.06
No	1.12 (0.61-2.03)	2.58 (1.40-4.73)		0.94 (0.51-1.75)	2.25 (1.21-4.17)		1.15 (0.63-2.12)	2.58 (1.40-4.74)	
**Quality of sex education received through school**									
Good quality	1.00	1.00	0.15	1.00	1.00	0.13	1.00	1.00	0.16
Poor quality	0.83 (0.51-1.36)	0.68 (0.35-1.35)		0.81 (0.49-1.33)	0.71 (0.36-1.41)		0.81 (0.49-1.32)	0.68 (0.34-1.34)	
No sex education	1.04 (0.56-1.94)	2.27 (1.19-4.33)		0.87 (0.45-1.66)	2.01 (1.05-3.88)		1.06 (0.56-1.99)	2.27 (1.19-4.33)	
**Usefulness of sex education**									
Useful	1.00	1.00	0.16	1.00	1.00	0.13	1.00	1.00	0.17
Not useful	1.96 (0.88-4.37)	2.13 (0.77-5.87)		2.03 (0.90-4.56)	2.71 (0.95-7.75)		1.83 (0.82-4.08)	2.10 (0.76-5.79)	
No sex education	1.18 (0.65-2.16)	2.79 (1.50-5.18)		1.00 (0.54-1.86)	2.46 (1.31-4.63)		1.21 (0.66-2.22)	2.79 (1.50-5.18)	

Hospital controls, complete case analysis (Total *N*=681; Females *N*=402, Males *N*=279)

Model 1: Adjusted for sex, age and religion

Model 2: Adjusted for sex, age, religion, highest educational attainment of either parent

Model 3: Adjusted for sex, age, religion, marital status

*OR* Odds Ratio (if the OR>1, this suggests that exposed individuals were more likely to have self-poisoned than non-exposed individuals)

*CI* Confidence Interval (if the CI overlaps 1, this suggests that there is no statistical evidence of a difference in risk between exposed and non-exposed individuals)

*p* values are presented for the test of interaction by sex

Sex -stratified models suggest that the effect sizes were overall greater among males, but formal tests of interaction found only weak evidence of an interaction by sex for the association between self-poisoning and receipt of sex education (*p*=0.06) (Table 4). No evidence was found of an interaction by sex for the associations between self-poisoning and quality (*p*=0.15) or usefulness (*p*=0.16) of sex education.

### Post-hoc analysis

The addition of marital status to the models (Model 3, Tables 3 and 4) did not alter the associations.

### Sensitivity analyses

When including all participants, regardless of missing data (Supplementary Tables 2 and 3), effect sizes for the associations between both receipt and usefulness of sex education and self-poisoning were larger but consistent with the main analysis using only complete cases. When using the community control series (Supplementary Tables 4 and 5), effect sizes for the associations between both receipt and usefulness of sex education and self-poisoning were larger but consistent with the main analysis using the hospital controls.

### Potential mediating factors

Supplementary Table 6 reports *number of children, domestic violence* and *childhood sexual abuse*, stratified by receipt of sex education and case-control status. Observation of the distributions of these variables did not highlight any potential mediators of the association between sex education and self-poisoning.

## Discussion

In this explorative analysis, we found that participants who reported not receiving sex education were more likely to have self-poisoned than those who did receive sex education, with nearly a doubling in risk. We also found strong evidence that those who received sex education but did not consider it useful, were more likely to have self-poisoned than those who found it useful. We found no statistical evidence that the quality of sex education, as measured here, was associated with self-poisoning risk.

In contrast to previous qualitative studies that have highlighted the links between social norms of sexuality and suicidal behaviour as an issue primarily among young women [12–14], we did not find statistical evidence of a difference between sexes. This may be because the sex education questions asked in the interview were culturally unacceptable, as women are not expected to have knowledge of sex before marriage [17]. Furthermore, gender-based cultural norms that dictate women should be modest and not discuss sexual matters may have affected women’s responses to the questions [13, 14, 22, 49]. It is possible that female participants were less likely than males to discuss sex education or openly critisise any sex education they received with the study researchers.

### Possible mechanisms and implications

By the WHO definition of CSE, the role of sex education is more than to prevent unwanted pregnancies and sexually-transmitted infections, but to equip young people with knowledge of wider reproductive health and relationship issues [31]. CSE could provide a platform for more open and earlier conversations around relationships and rights, including domestic violence. There is strong evidence that school-based sex education programmes have the potential to improve knowledge, change attitudes and reduce the incidence of domestic and intimate partner violence [29]. Mechanisms for how this works include providing conflict management skills, shifting gender norms, and focusing on social justice [29]. Exposure to domestic violence has been identified as a risk factor for suicidal behaviour in a variety of contexts [50–52], including Sri Lanka [12, 45, 53]. A plausible hypothesis is that prevention of domestic violence may be one mediating pathway between sex education and reduced deliberate self-poisoning in Sri Lanka. Another potential mediating pathway is through preventing childhood sexual abuse [29, 54, 55]. We explored whether there was any evidence that domestic violence or childhood sexual abuse were mediating factors in our sample by presenting the distributions of these variables by both self-poisoning and receipt of sex education (Supplementary Table 6). When crudely observing these distributions, there was no indication that these were potential mediators. However, this was likely due to methodological issues as the study was not designed to explore this question, and should therefore not be interpreted as lack of an association (see further information in Supplementary Table 6 footnotes).

Teaching on the biological aspects of puberty may also be facilitated through CSE. In some Sri Lankan communities, puberty is a taboo subject that, like sex, is not openly discussed in the home. Puberty is seen as a time when young people, especially girls, are expected to develop a sense of shame, shyness, respect and compliance towards their parents [19]. As a result, young people feel they cannot ask parents about what is happening to their bodies and parents feel that, like sex education, their children should learn about it through other sources such as school [19]. A study conducted by the United Nations Population Fund found that 66% of girls were not aware of menstruation until their first period and 75% believed menstrual blood was polluted [54]. Yet, puberty also represents a time of celebration; rituals such as “big girl parties” (arranged when girls reach menarche) are ubiquitous across most ethnic and cultural groups in some form [18, 19]. Perhaps this discordance explains why issues related to puberty have been identified as a source of psychological distress among young people in Sri Lanka [56].

Reproductive consequences such as large numbers of children, inadequate birth spacing, unplanned pregnancies, and abortions, may also be mechanisms through which lack of effective sex education contributes towards self-harm. Our study did not collect data on these factors other than number of children, which did not appear to have a role in explaining the association between sex education and self-poisoning in our sample (Supplementary Table 6). Again, this is likely due to methodological issues as opposed to evidence of lack of an association.

Finally, as a public health intervention that has the potential for widespread coverage, CSE may help to shape and change gender norms around sexual behaviour over time. There are ample examples from different settings that demonstrate how gender norms influence health [57]. For example, in Zambia, discordance between the attitudes of women to pre-marital sex and women’s sexual behaviour is strongly correlated with the prevalence of HIV [57]. In some Sri Lankan communities, if a girl is seen ‘with’ a boy in public, even if not in a romantic sense, she is seen to have brought shame upon herself and by extension her family [14, 21]. In this sense, it is not the relationship itself that leads to the ‘suicide-like act’, but the parental reaction to the discovery of an illicit relationship, the associated shame for her and her family, fear of the repercussions, and actual repercussions, that lead to self-poisoning. Over 90% of our sample reported that their parents did not understand their worries when they were a child (Table 1), which suggests a significant degree of intergenerational dissonance. There is some evidence from the US that school-based relationship education can lead not only to improved dating relationships, but also improved parent-adolescent relationships [58]. However, this may not be translatable to the Sri Lankan context. An appropriate future research area would be to explore what schools can do to equip Sri Lankan adolescents with resilience to gender-based cultural norms and family expectations surrounding sex and relationships, and whether this can be incorporated into a CSE curriculum.

The social sciences literature presents two main schools of thought on how changes to societal norms can be achieved; changing individuals’ attitudes, or changing the norms embedded within institutions, which ultimately requires a shift in power structures [59]. The possible mechanisms outlined above mostly align with the assumption that changing individual attitudes around sexual behaviour will lead to a reduction in self-poisoning. However, recent backlash at the suggestion of introducing a sex education textbook in Sri Lanka [37] demonstrated that changes in attitude are required at an institutional level. Those working in education policy must be motivated to prioritise the delivery of high-quality, age-appropriate sex education in schools, and have the necessary skills to do so.

Sex education is, in theory, easily modifiable at a population-level. For sex education to be a target for intervention, it is essential that stakeholders such as teachers and parents are engaged. Two thirds of both cases and controls in our sample agreed that their teachers had been interested in them and their life, not just schoolwork (Table 1), which suggests a degree of pastoral involvement and a sense that teachers are interested in students’ wellbeing. However, there exist several barriers to implementing sex education in schools, such as perceived abilities of staff, uncertainties around roles, fears of encouraging precocious sexual behaviour, threats from parents and shyness and anxieties of teachers over talking about sex related topics to children [35, 36, 54]. For CSE to be implemented and achieve widespread coverage, teachers need to feel empowered to discuss sex with their students. This can only happen with adequate teacher training and mentoring, supported by administrative and policy change, and crucially with student and community buy-in. NGOs which are already skilled in delivering CSE using participatory methods and have readily-available educational materials may be well-placed to support the training of teachers [60].

Given the multifactorial nature of self-harm, we suggest that the findings of this explorative analysis should be applied not on an individual, but a population level. One explanatory model is that the point at which sex education is delivered in schools is an opportune time to intervene in the lives of young people, by providing them with knowledge, which in turn empowers them, leading to population-level change in societal attitudes and behaviours. This type of change undoubtedly takes time. Future research, conducted over a long period of time, is required to explore whether this theory holds true.

### Strengths and limitations

To our knowledge, this is the first study to quantitatively explore the association between sex education and self-poisoning in Sri Lanka. This is important, in light of evidence that suggests that mental illness is less prevalent among those who self-harm in LMICs compared to HICs, suggesting that other risk factors are likely to play a more significant role [8–10, 61]. The findings add to the field in terms of understanding the life-course trajectory of self-harm in Sri Lanka and provide an important starting point for future research exploring this area in-depth. The variables had few missing data points and running the analyses with all participants (irrespective of missing data) did not change the overall interpretation of the results.

The findings of this study should be interpreted in light of its limitations. First, the sex education questions were not validated, therefore it is not certain whether they accurately captured the concepts of receipt, quality and usefulness of sex education in this context. The questions did not specify where or when sex education was received, or what topics participants considered to be sex education (for example, basic anatomical knowledge or a much wider range of topics such as those promoted by the WHO [31]). The participants may not have known what constitutes good quality or useful sex education if they had nothing to compare their experiences to and may have been unfamiliar with the concept of evaluating teaching sessions. Furthermore, the questions were potentially not culturally sensitive or acceptable. There were also potential procedural issues. The study team conducting interviews reported that participants seemed hesitant to answer these questions and researchers often had to repeat them several times. Gender-matching the data collectors to interviewees may have improved disclosure, however we do not think it would have made a significant difference as all data collectors were female, men in this context generally find it easier to talk about sex than women, and our stratified analysis suggested that disclosure among men was not an issue, with higher effect sizes seen among males. The researchers also raised issues with the placement of the sex education questions in the interview, as they appeared relatively early on in the encounter, before good rapport was established. All of these factors suggest our findings are likely to underestimate the true proportion of the population who did not receive, or received poor quality/not useful, sex education. Another limitation is that data was not collected on potentially important mediating factors such as number of pregnancies, miscarriages, stillbirths and abortions. Finally, significant regional variations in both suicide rates [62] and reproductive health indices [63] exist in Sri Lanka, therefore these findings may not be generalisable nationally.

## Conclusion

This study reports novel findings of an association between sex education and self-poisoning in Kandy, Sri Lanka, and provides a starting point for further work in this area. As sex education can be modified at a population level, this association should be explored further to predict whether improvements in sex education are associated with a reduction in self-poisoning. Future studies would ideally be prospective in nature, with the caveat that self-poisoning is a rare outcome which may not be amenable to prospective study designs. There is a need for a validated tool to measure experiences of sex education in Sri Lanka. Future research should also aim to understand the theory of change in more depth, which would be best achieved by conducting qualitative research with young people, parents and teachers delivering sex education.

## Supplementary Information

**Additional file 1.** Supplementary tables, referred to in the main manuscript

## Data Availability

Data are available at the University of Bristol data repository, data.bris, at 10.5523/bris.37pg6mv6x35r12b98aoq4blcgs. Given the sensitivity of the data and only researchers at verifiable institutions will be able to access data. Any requests will be reviewed by the University of Bristol Access Committee, which includes senior researchers and representatives from the University. Data will only be released once a controlled data access agreement has been signed by a nominated institutional signatory.

## Abbreviations

A/L: Advanced level
CI: Confidence interval
CSE: Comprehensive sexuality education
HICs: High-income countries
IQR: Interquartile range
LGBTQ+: Lesbian, gay, bisexual, transgender, queer +
LMICs: Low- and middle-income countries
NGOs: Non-governmental organisations
O/L: Ordinary level
OR: Odds ratio
THP: Teaching Hospital Peradeniya
US: United States
WHO: World Health Organisation
