# Supplementary material for: Sex education and self-poisoning in Sri Lanka: an explorative analysis
Source: BMC Public Health. 2022 Jan 6;22:26. doi: 10.1186/s12889-021-12374-4 (PMC8740467; doi:10.1186/s12889-021-12374-4)
Supplement: Supplementary file 1 — Additional file 1. Supplementary tables, referred to in the main manuscript [file 12889_2021_12374_MOESM1_ESM.docx]

**Supplementary Materials**

Article title: Sex education and self-poisoning in Sri Lanka: an explorative analysis

**Supplementary table 1: Sex and age-adjusted (“crude”) associations between sex education and self-poisoning**

|  | **Cases**  n (%)  N = 236 | **Hospital controls**  n (%)  N = 445 | **Sex and age-adjusted (“crude”) associations**  OR (95% CI) |
| --- | --- | --- | --- |
| **Receipt of sex education** |  | | |
| Yes | 167, 70.8 (64.6-76.2) | 352, 79.1 (25.1-82.6) | 1.00 |
| No | 69, 29.2 (23.8-35.4) | 93, 20.9 (17.4-24.9) | 1.94 (1.29-2.90) |
| **Quality of sex education received through school** |  | | |
| Good quality | 113, 47.9 (41.6-54.3) | 211, 47.4 (42.8-52.1) | 1.00 |
| Poor quality | 54, 22.9 (18.0-28.7) | 141, 31.7 (27.5-31.2) | 0.73 (0.50-1.08) |
| No sex education | 69, 29.2 (23.8-35.4) | 93, 20.9 (17.4-24.9) | 1.72 (1.12-2.65) |
| **Usefulness of sex education** |  | | |
| Useful | 146, 61.9 (55.5-67.9) | 328, 73.7 (69.4-77.6) | 1.00 |
| Not useful | 21, 8.9 (5.9-13.3) | 24, 5.4 (3.6-7.9) | 1.94 (1.04-3.60) |
| No sex education | 69, 29.2 (23.8-35.4) | 93, 20.9 (17.4-24.9) | 2.06 (1.36-3.10) |

Hospital controls, complete case analysis (Total N=681)

OR: Odds Ratio (if the OR>1, this suggests that exposed individuals were more likely to have self-poisoned than non-exposed individuals)

CI: Confidence Interval (if the CI overlaps 1, this suggests that there is no statistical evidence of a difference in risk between exposed and non-exposed individuals)

**Supplementary table 2**: **Multivariable logistic regression models for associations between sex education and self-poisoning**

|  | **Model 1** | | **Model 2** | | | **Model 3** | |
| --- | --- | --- | --- | --- | --- | --- | --- |
|  | OR (95% CI) | n | OR (95% CI) |  | n | OR (95% CI) | n |
| **Receipt of sex education** |  | |  | | |  | |
| Yes | 1.00 | 793 | 1.00 | 699 | | 1.00 | 793 |
| No | 2.03 (1.40-2.93) |  | 1.48 (0.97-2.26) |  |  | 2.04 (1.41-2.95) |  |
| **Quality of sex education received through school** |  | |  | | |  | |
| Good quality | 1.00 | 775 | 1.00 | 681 | | 1.00 | 775 |
| Poor quality | 0.75 (0.52-1.10) |  | 0.77 (0.52-1.16) |  |  | 0.75 (0.51-1.09) |  |
| No sex education | 1.79 (1.21-2.67) |  | 1.32 (0.84-2.08) |  |  | 1.80 (1.21-2.68) |  |
| **Usefulness of sex education** |  | |  | | |  | |
| Useful | 1.00 | 791 | 1.00 | 697 | | 1.00 | 791 |
| Not useful | 1.90 (1.04-3.45) |  | 2.07 (1.10-3.90) |  |  | 1.89 (1.04-3.44) |  |
| No sex education | 2.12 (1.46-3.08) |  | 1.56 (1.02-2.38) |  |  | 2.13 (1.46-3.09) |  |

Hospital controls, all subjects regardless of missingness

Model 1: Adjusted for sex, age and religion

Model 2: Adjusted for sex, age, religion, highest educational attainment of either parent

Model 3: Adjusted for sex, age, religion, marital status

OR: Odds Ratio (if the OR>1, this suggests that exposed individuals were more likely to have self-poisoned than non-exposed individuals)

CI: Confidence Interval (if the CI overlaps 1, this suggests that there is no statistical evidence of a difference in risk between exposed and non-exposed individuals)

**Supplementary table 3: Multivariable logistic regression models for associations between sex education and self-poisoning, stratified by sex**

|  | **Model 1** | | | **Model 2** | | | **Model 3** | | |
| --- | --- | --- | --- | --- | --- | --- | --- | --- | --- |
|  | **Female** | **Male** | p | **Female** | **Male** | p | **Female** | **Male** | p |
|  | OR (95% CI) | OR (95% CI) |  | OR (95% CI) | OR (95% CI) |  | OR (95% CI) | OR (95% CI) |  |
| **Receipt of sex education** |  | | |  | | |  | | |
| Yes | 1.00 | 1.00 | 0.11 | 1.00 | 1.00 | 0.06 | 1.00 | 1.00 | 0.12 |
| No | 1.46 (0.85-2.51) | 2.79 (1.66-4.71) |  | 1.02 (0.55-1.88) | 2.14 (1.17-3.92) |  | 1.51 (0.88-2.60) | 2.78 (1.64-4.69) |  |
| n | n=451 | n=342 |  | n=410 | n=289 |  | n=451 | n=342 |  |
| **Quality of sex education received through school** |  | | |  | | |  | | |
| Good quality | 1.00 | 1.00 | 0.18 | 1.00 | 1.00 | 0.13 | 1.00 | 1.00 | 0.19 |
| Poor quality | 0.84 (0.52-1.35) | 0.60 (0.32-1.13) |  | 0.81 (0.49-1.33) | 0.71 (0.36-1.41) |  | 0.81 (0.50-1.31) | 0.59 (0.31-1.12) |  |
| No sex education | 1.27 (0.71-2.25) | 2.44 (1.38-4.31) |  | 0.87 (0.45-1.66) | 2.01 (1.05-3.88) |  | 1.29 (0.72-2.31) | 2.41 (1.37-4.27) |  |
| n | n=443 | n=332 |  | n=402 | n=279 |  | n=443 | n=332 |  |
| **Usefulness of sex education** |  | | |  | | |  | | |
| Useful | 1.00 | 1.00 | 0.28 | 1.00 | 1.00 | 0.16 | 1.00 | 1.00 | 0.29 |
| Not useful | 2.07 (0.96-4.47) | 1.85 (0.71-4.79) |  | 1.89 (0.85-4.20) | 2.72 (0.95-7.74) |  | 1.96 (0.90-4.26) | 1.80 (0.69-4.67) |  |
| No sex education | 1.54 (0.89-2.66) | 2.91 (1.71-4.96) |  | 1.07 (0.58-1.98) | 2.30 (1.24-4.25) |  | 1.58 (0.91-2.74) | 2.89 (1.69-4.93) |  |
| n | n=450 | n=341 |  | n=409 | n=288 |  | n=450 | n=341 |  |

Hospital controls, all subjects regardless of missingness

Model 1: Adjusted for sex, age and religion

Model 2: Adjusted for sex, age, religion, highest educational attainment of either parent

Model 3: Adjusted for sex, age, religion, marital status

OR: Odds Ratio (if the OR>1, this suggests that exposed individuals were more likely to have self-poisoned than non-exposed individuals)

CI: Confidence Interval (if the CI overlaps 1, this suggests that there is no statistical evidence of a difference in risk between exposed and non-exposed individuals)

p values are presented for the test of interaction by sex

**Supplementary table 4**: **Multivariable logistic regression models for associations between sex education and self-poisoning**

|  | **Model 1**  OR (95% CI)  N=620 | **Model 2**  OR (95% CI)  N=620 | **Model 3**  OR (95% CI)  N=620 |
| --- | --- | --- | --- |
| **Receipt of sex education** |  | | |
| Yes | 1.00 | 1.00 | 1.00 |
| No | 2.78 (1.77-4.38) | 2.43 (1.53-3.87) | 2.79 (1.77-4.39) |
| **Quality of sex education received through school** |  | | |
| Good quality | 1.00 | 1.00 | 1.00 |
| Poor quality | 0.60 (0.40-0.89) | 0.57 (0.38-0.86) | 0.60 (0.40-0.89) |
| No sex education | 2.28 (1.42-3.67) | 1.94 (1.19-3.17) | 2.28 (1.42-3.68) |
| **Usefulness of sex education** |  | | |
| Useful | 1.00 | 1.00 | 1.00 |
| Not useful | 2.70 (1.34-5.42) | 2.96 (1.45-6.04) | 2.69 (1.34-5.42) |
| No sex education | 3.02 (1.91-4.79) | 2.65 (1.66-4.24) | 3.03 (1.91-4.79) |

Community controls, complete case analysis (Total N=620)

Model 1: Adjusted for sex, age and religion

Model 2: Adjusted for sex, age, religion, highest educational attainment of either parent

Model 3: Adjusted for sex, age, religion, marital status

OR: Odds Ratio (if the OR>1, this suggests that exposed individuals were more likely to have self-poisoned than non-exposed individuals)

CI: Confidence Interval (if the CI overlaps 1, this suggests that there is no statistical evidence of a difference in risk between exposed and non-exposed individuals)

**Supplementary table 5**: **Multivariable logistic regression models for associations between sex education and self-poisoning, stratified by sex**

|  | **Model 1** | | | **Model 2** | | | **Model 3** | | |
| --- | --- | --- | --- | --- | --- | --- | --- | --- | --- |
|  | **Female** | **Male** | p | **Female** | **Male** | p | **Female** | **Male** | p |
|  | OR (95% CI) | OR (95% CI) |  | OR (95% CI) | OR (95% CI) |  | OR (95% CI) | OR (95% CI) |  |
| **Receipt of sex education** |  | | |  | | |  | | |
| Yes | 1.00 | 1.00 | 0.08 | 1.00 | 1.00 | 0.08 | 1.00 | 1.00 | 0.07 |
| No | 1.95 (1.04-3.66) | 4.09 (2.09-8.03) |  | 1.66 (0.87-3.19) | 3.63 (1.83-7.19) |  | 1.95 (1.04-3.66) | 4.16 (2.11-8.20) |  |
| **Quality of sex education received through school** |  | | |  | | |  | | |
| Good quality | 1.00 | 1.00 | 0.15 | 1.00 | 1.00 | 0.14 | 1.00 | 1.00 | 0.14 |
| Poor quality | 0.68 (0.42-1.10) | 0.48 (0.24-0.96) |  | 0.66 (0.40-1.08) | 0.44 (0.22-0.90) |  | 0.68 (0.41-1.10) | 0.47 (0.24-0.95) |  |
| No sex education | 1.67 (0.87-3.22) | 3.06 (1.56-5.94) |  | 1.41 (0.71-2.77) | 2.60 (1.25-5.41) |  | 1.67 (0.87-3.22) | 3.11 (1.51-6.38) |  |
| **Usefulness of sex education** |  | | |  | | |  | | |
| Useful | 1.00 | 1.00 | 0.18 | 1.00 | 1.00 | 0.17 | 1.00 | 1.00 | 0.18 |
| Not useful | 2.36 (0.99-5.61) | 3.41 (1.03-11.34) |  | 2.47 (1.02-5.99) | 4.14 (1.22-14.05) |  | 2.37 (1.00-5.66) | 3.38 (1.01-11.25) |  |
| No sex education | 2.06 (1.09-3.88) | 4.67 (2.34-9.33) |  | 1.76 (0.91-3.38) | 4.17 (2.07-8.42) |  | 2.06 (1.09-3.88) | 4.72 (2.36-9.47) |  |

Community controls, complete case analysis (Total N=620; Females N=369, Males N=251)

Model 1: Adjusted for sex, age and religion

Model 2: Adjusted for sex, age, religion, highest educational attainment of either parent

Model 3: Adjusted for sex, age, religion, marital status

OR: Odds Ratio (if the OR>1, this suggests that exposed individuals were more likely to have self-poisoned than non-exposed individuals)

CI: Confidence Interval (if the CI overlaps 1, this suggests that there is no statistical evidence of a difference in risk between exposed and non-exposed individuals)

p values are presented for the test of interaction by sex

**Supplementary table 6: Distribution of potential mediators of the association between sex education and self-poisoning, stratified by receipt of sex education and case-control status**

|  | **Did not receive sex education**  n (%)  Total N=212 | | **Received sex education**  n (%)  Total N=581 | |
| --- | --- | --- | --- | --- |
|  | **Cases**  n (%)  N=104 | **Hospital controls**  n (%)  N=108 | **Cases**  n (%)  N=191 | **Hospital controls**  n (%)  N=390 |
| **Number of children** |  | | | |
| 0 | 37 (35.6) | 32 (29.6) | 122 (63.9) | 272 (69.7) |
| 1-2 | 37 (35.6) | 42 (38.9) | 54 (28.3) | 92 (23.6) |
| 3-6 | 30 (28.8) | 34 (31.5) | 15 (7.9) | 26 (6.7) |
| Missing | 0 (0.0) | 0 (0.0) | 0 (0.0) | 0 (0.0) |
| **Domestic violence** |  | | | |
| Yes | 35 (33.7) | 20 (18.5) | 92 (48.2) | 71 (18.2) |
| No | 66 (63.5) | 86 (79.6) | 99 (51.8) | 313 (80.3) |
| Missing | 3 (2.9) | 2 (1.9) | 0 (0.0) | 6 (1.5) |
| **Childhood sexual abuse** |  | | | |
| Yes | 10 (9.6) | 8 (7.4) | 21 (11.0) | 36 (9.2) |
| No | 90 (86.5) | 98 (90.7) | 170 (89.0) | 351 (90.0) |
| Missing | 4 (3.8) | 2 (1.9) | 0 (0.0) | 3 (0.8) |

Hospital controls, all subjects regardless of missingness (Total N = 793)

**Supplementary table 6 footnotes: discussion on potential mediators**

- **Domestic violence:** The prevalence of domestic violence found in our sample was in-keeping with that found in other studies (1,2). This is suggestive that non-disclosure of domestic violence was not a major issue among our sample.
- **Childhood sexual abuse:** The prevalence of childhood sexual abuse is discussed in another analysis derived from this same case-control study (3). The authors note that the prevalence of childhood abuse was lower than expected. They highlight several potential reasons for this, including the use of a severity threshold to identify levels of abuse and the face-to-face nature of the interviews, meaning that participants may have been reluctant to report experience of childhood sexual abuse.

References:

1. DCS. (2016). *Sri Lanka Demographic and health survey 2016*. Colombo, Sri Lanka: Department of Census and Statistics (DCS) and Ministry of Health, Nutrition and Indigenous Medicine.
2. Guruge, S., Jayasuriya-Illesinghe, V., Gunawardena, N., & Perera, J. (2015). Intimate partner violence in Sri Lanka: A scoping review. *Ceylon Medical Journal*, 60(4), 133–138. doi: 10.4038/cmj.v60i4.8100
3. Rajapakse T, Russell AE, Kidger J, Bandara P, López-López JA, Senarathna L, et al. (2020) Childhood adversity and self-poisoning: A hospital case control study in Sri Lanka. PLoS ONE 15(11): e0242437. https://doi.org/10.1371/journal.pone.0242437
